# Identifying Currents in the Gene Pool for Bacterial Populations Using an Integrative Approach

**DOI:** 10.1371/journal.pcbi.1000455

**Published:** 2009-08-07

**Authors:** Jing Tang, William P. Hanage, Christophe Fraser, Jukka Corander

**Affiliations:** 1 Department of Mathematics and Statistics, University of Helsinki, Helsinki, Finland; 2 Department of Infectious Disease Epidemiology, Imperial College London, London, United Kingdom; 3 Department of Mathematics, Åbo Akademi University, Åbo, Finland; University of California San Diego, United States of America

## Abstract

The evolution of bacterial populations has recently become considerably better understood due to large-scale sequencing of population samples. It has become clear that DNA sequences from a multitude of genes, as well as a broad sample coverage of a target population, are needed to obtain a relatively unbiased view of its genetic structure and the patterns of ancestry connected to the strains. However, the traditional statistical methods for evolutionary inference, such as phylogenetic analysis, are associated with several difficulties under such an extensive sampling scenario, in particular when a considerable amount of recombination is anticipated to have taken place. To meet the needs of large-scale analyses of population structure for bacteria, we introduce here several statistical tools for the detection and representation of recombination between populations. Also, we introduce a model-based description of the shape of a population in sequence space, in terms of its molecular variability and affinity towards other populations. Extensive real data from the genus *Neisseria* are utilized to demonstrate the potential of an approach where these population genetic tools are combined with an phylogenetic analysis. The statistical tools introduced here are freely available in BAPS 5.2 software, which can be downloaded from http://web.abo.fi/fak/mnf/mate/jc/software/baps.html.

## Author Summary

The study of bacterial population biology is complicated by the fact that, although bacteria are largely asexual, they can also exchange genetic materials through homologous recombination. Unlike eukaryotes, recombination in bacteria is not an obligatory process. Furthermore, the recombination mechanisms are subject to many biological and ecological factors that can vary even within different populations of the same species. Although increasing evidence for homologous recombination has been found in many bacterial species, determining the frequency of recombination and understanding the influence that it exerts upon the evolution of bacterial populations remains a challenging work. In this article, we provide a dynamic picture of recombination within and between closely related bacteria species. Through an integration of several Bayesian statistical models, our method highlights the importance of a quantitative estimation of recombination. Our analyses of a challenging multi-locus sequence typing (MLST) database demonstrate that combined analyses using both traditional phylogenetic methods, explorative MLST tools and Bayesian population genetic models can together yield interesting biological insights that cannot easily be reached by any of the approaches alone.

## Introduction

It has become increasingly evident that recombination plays a major role in shaping the genetic structure of bacterial populations. Whether or not certain populations (as defined by allele frequencies) are more likely than others to undergo recombination, either as donors or recipients of DNA, is not well understood, though there are several biological reasons why this might be the case. Such preferential recombination, which we may intuitively describe as *currents in the gene pool* [1], should lead to a greater degree of admixture between the populations in question, and this should be detectable using DNA sequence data. Conceptually related investigation of highways of gene sharing among bacterial species at a general level was done by [2], who found evidence for uneven distribution of transfer intensity among groups of prokaryotes.

Discovery of such gene flow currents is scientifically interesting in its own right, as a means for characterizing populations and reflecting upon accumulated taxonomic understanding of their heterogeneity. However, there are other potential uses for detailed knowledge concerning the genetic structure of a bacterial population, e.g. when it can be connected to patterns of virulence and antibiotic resistance.

Statistical analysis of molecular variation and reproductive isolation in natural populations is in many cases far more challenging for bacteria than for eukaryotic organisms, due to difficulties in acquiring broad-coverage samples and the putatively complex admixture events [3]. Traditional population genetic tools for inferring genetic barriers within a population, such as 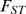 measures [4], are not usually applicable to bacterial molecular data given the lack of relevant populations to condition the calculations on, albeit some exceptions exist (see, e.g. [5]). Standard phylogenetic analyses, on the other hand, may provide a distorted view of the ancestral relationships among bacteria when recombination events are sufficiently common in a population. Moreover, they do not yield a detailed and easily interpretable picture of the patterns of admixture and eventual genetic barriers, as such constructs are not present in the standard phylogenetic models that can be routinely applied to large data sets. However, an algorithmic approach to phylogenetic analysis which can build networks for hundreds of taxa and can be useful for data sets harbouring recombination was introduced by [6]. A model-based phylogenetic method (ClonalFrame) that deals explicitly with recombinations was introduced by [7], however, it does not easily scale up to the level of population complexity we are here interested in, due to the extreme computational intensity of the model fitting for large databases.

With the above-mentioned difficulties, it is hardly surprising that a Bayesian statistical approach based on explicit admixture models has recently gained popularity in studies of bacterial populations [8],[9]. Such models are anchored in the general idea of a probabilistic partition, where an unknown origin of an arbitrary quantity (for example, the membership of an individual) is inferred through the conditional probability of the origin over the range of putative alternatives (commonly referred to as clusters), given the observed features of the quantity. Application of such partition models has been made possible by a class of generic Markov chain Monte Carlo (MCMC) algorithms [10], that can be used for fitting the models to molecular data.

Despite the success of the standard MCMC approach in a variety of studies of bacterial populations (see e.g. [11]), it is clear, both theoretically and practically, that the performance of the standard MCMC computation decreases rapidly as the complexity of the estimated population structure and the size of the investigated data set increases [10],[12]. To address this, an array of methods has been introduced and implemented in the software BAPS [13]–[16]. Here we introduce a graphical characterization of recombination patterns from MLST data using a weighted network with statistically identified populations as cluster nodes and estimated average levels of DNA transition as relative gene flow weights. Also, we introduce a model-based representation of the molecular variability of populations and their affinities towards each other. We refer to this as the *genetic shape* of an identified population. However, it is important to notice that a population identified by BAPS may have a different interpretation in different evolutionary contexts. The BAPS models target for identifying molecular evidence that links a particular group of strains together in terms of sufficiently similar nucleotide frequencies. Thus, such a population may for instance arise in the analysis due to common ancestry within a clonal complex. In contrast, a population can also be identified from the traces left by recombination events which have imposed considerable gene flow between separate lineages of strains. Also, under certain circumstances a more heterogeneous population may arise analogously to long branch attraction in phylogenetics, in particular, when very limited numbers of strains from the corresponding lineages are present among the analyzed samples. All these three cases are illustrated in our analyses. As BAPS is capable of capturing a variety of distinct biological signals hidden in molecular data, interpretation of the identified populations must be done with care, using preferably both complementary phylogenetic methods and auxiliary knowledge about the strains under investigation.

To illustrate the levels of complexity at which our methods can operate, we consider a population sample of 5086 strains that have been identified as *Neisseria meningiditis* and *Neisseria lactamica* species. We also present analyses of simulated data to demonstrate the potential of our Bayesian approach to handle large databases and complex genetic population structures. Our analyses illustrate that biological insights to complex data are best gained by combining several complementary methods of analysis.

## Materials and Methods

### A stochastic model of gene flow in bacterial populations

Assume that the target population consists of 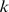 genetically distinct populations, among which the extent of gene flow is to be modeled. Usually, 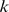 and the genetic population structure associated with it are *a priori* unknown. In our statistical approach presented later we consider in detail the inference of these from molecular data. Here we aim to estimate the strength of gene flow via a stochastic characterization of the rates of admixture between the 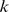 identified populations.

Let 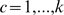, index the 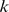 populations and let 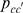 represent for the population 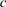 the probability of an strain acquiring DNA from bacteria present in the population 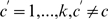. DNA acquisition could be understood as an aggregated result of the currently known mechanisms (conjugation, transduction and transformation). Conditional on the probability 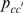, it is possible to consider a sample of 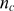 unrelated strains from population 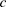 to represent 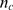 Bernoulli trials, where the binary outcome 
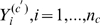
, refers to the success/failure of DNA acquisition from this particular source. These are obviously considerably simplifying assumptions, but they allow us to characterize patterns of admixture. Were the outcomes 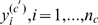, known, the relative admixture could simply be characterized by 
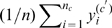
. However, we note that 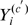 in reality represents intrinsically unobservable latent events during some interval of the evolutionary time scale under consideration.

Assuming that a particular strain within population 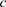 has acquired DNA from the population 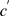 (i.e. 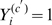), we may attempt to quantify the intensity with which such events have occurred over the analysed sequence. A multitude of statistical break-point models designed to capture such recombination traces have been introduced in the literature, e.g. [17]–[19]. For such models the focus has typically been on a small number of short viral genomes, to identify the locations where putative recombination events have taken place. In the most basic form, recombination may be represented by a homogeneous spatial Poisson process 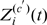, where the events correspond to the number of recombinations within the genome of an strain 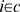, such that the DNA is acquired from the population 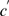. It follows for such a process that the stochastic variable 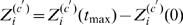, with 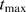 equal to the total length of the considered sequence, has the Poisson distribution
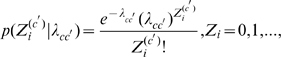
(1)where 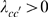 represents the average rate of events in which DNA is imported from population 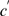 to 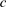. Again, if the outcomes 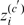 were observed, the average rate could be statistically quantified, e.g. as 
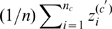
, by using the maximum likelihood estimate.

To arrive at a statistical characterization of the rates of admixture among the populations under the above framework, let 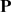 denote a 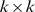 matrix of probabilities, such that the element 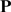 equals 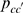. Further, let the 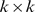 matrix 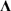, with the elements 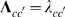, represent collectively the Poisson intensities. Let 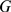 be a directed graph with the 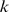 populations as the node set 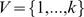, and 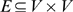 as the arc set. Each arrow 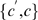 in 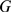 can now be associated with a weight 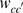 depicting the rate of admixture from 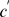 to 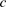. For instance, a gene flow weight matrix 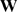 can be defined in terms of the elementwise matrix product 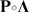, with the convention that the diagonal elements 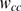 are normalized by the other elements on 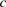
th row of 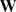. When an element 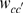 equals zero, it is natural to set 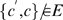, i.e. the corresponding arrow is absent in 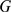.

It follows from the definition of 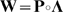 that these parameters remain unidentifiable when the events 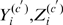 are unobserved, as a suitable rescaling of the model configuration can yield identical likelihoods. The statistical challenge related to this context is further accentuated by the fact that the underlying genetic structure, i.e. the number of underlying populations 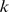 as well as their molecular characteristics, is unknown *a priori*. Modern Bayesian statistical framework utilizing state-of-the-art MCMC computation can in principle be thought to provide a suitable setting for fitting such models to MLST sequence data. However, the computational complexity associated with the models suggests that formal posterior inferences would remain beyond the bounds of computational tractability even for only moderately sized population samples. This is crucial, as to study such problems we require large samples with a broad coverage of the genetic variation in the underlying population. Therefore, we consider here an approximate inference strategy to estimate 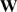, which is computationally manageable for large samples, while still providing a reasonable statistical characterization of parameters that can be interpreted in terms of 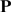
and 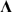 in the above model formulation.

### A Bayesian mixture model for the genetic structure of a population

Assume we have a sampled set of 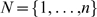 aligned DNA sequences 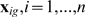, from 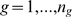 genomic regions in 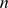 bacterial strains. A concatenated sequence for an strain 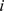 is denoted by 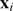 and 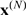 refers jointly to all the DNA sequence data from the 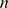 strains. For any subset 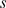 of strains from 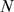, the notation 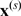 will be used for the DNA data observed for these strains.

Let 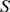 be a partition of the 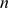 strains representing an underlying genetic structure (i.e. a representation of a genetic mixture model), with the clusters 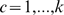 corresponding to genetically distinct populations. Hereafter we will use the terms ‘cluster’ and ‘population’ interchangeably. Mathematically, 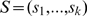 (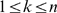) is a collection of subsets of 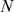, such that 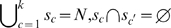, for all 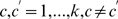. Symbol 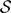 defines the space of all such partitions for a given 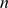. For any partition 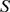, cardinalities of the populations are denoted by 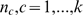.

In a series of earlier works in [12],[13] various stochastic partition models have been introduced for Bayesian inference about genetic population structure based on different types of molecular information. The mathematical motivation of the stochastic partition approach was recently derived by [20]. Under these models, the biological hypothesis corresponding to any particular partition 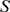, states that the strains allocated in the same cluster represent a sample from a genetically distinct population, and thus, the partition provides a qualitative representation of the underlying genetic population structure.

Let 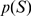 denote the *a priori* uncertainty about the underlying genetic structure in terms of a probability distribution over the space 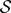. Then, we may specify the probability measure
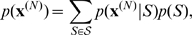
(2)where 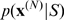 is the marginal likelihood of the sequence data given the structure. The posterior distribution of 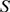 given the sequence data is determined by Bayes' rule according to
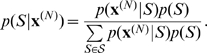
(3)


Here we use a Bayesian estimate of the genetic structure provided by the posterior mode
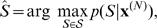
(4)or possibly separately for a range of such estimates identified by stochastic optimization, if the molecular data are not decisively supporting a single structure. Methods to numerically obtain such estimates have been introduced by [12],[20].

The marginal likelihood for the observed sequence data given any structure 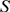 has under the stochastic partition framework the following product form
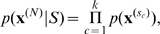
(5)which encapsulates a symmetry among the underlying populations 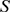, as any specific labeling of them without further auxiliary information would not be possible.

However, to explicitly specify the terms 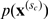 a number of assumptions are required. Here we exploit the genetic linkage model developed by [15] to provide an explicit characterization of the terms in (5) for MLST type sequence data. The linkage model captures dependencies in the sequence data in terms of a Markovian model for each gene, such that each population has its own nucleotide frequency parameters, the joint prior distribution of which factorizes according to the Markovian model. Utilizing the standard results for so called hyper-Markov probability laws for multinomial-Dirichlet distributions, it is possible to calculate the marginal likelihood analytically given any value of S. This result is of importance, because it enables the development of an efficient learning algorithm which avoids Monte Carlo errors associated with the nucleotide frequency parameters in the populations specified by the genetic structure model. However, it should be noted that because the genetic mixture model operates at the level of sequence data, it is vulnerable to misalignments of the sequences similar to other comparable statistical methods.

### Statistical characterization of admixture

Given a plausible representation of the underlying genetic population structure based on (4), our aim is to obtain a model-based characterization of the rates of admixture between the populations, such that an estimate may be derived for the gene flow weight matrix 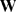. This sequential estimation strategy is motivated by the observation, that joint estimation of 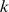 and the extent of admixture leads to problems with statistical identifiability and over-fitting discussed by [14]. In particular, they described a property of the admixture models which enables an increase in the number of populations without necessarily increasing the effective number of parameters (allele frequencies) in the model. This is in contrast with genetic mixture models, where such an increase always occurs as a function of the number of populations, thus resolving the problem with weak identifiability and/or high dependence of the inferences on the particular prior distribution used in the analysis.

The most recent version of the BAPS software (5.2) contains an implementation of the admixture estimation algorithm introduced by [16] under the linkage model of [15]. Here we use this procedure to estimate the extent of admixture among the populations.

Let 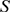 be an estimate of the genetic structure underlying the sample according to (4), and let 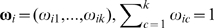, be a vector of admixture coefficients representing the proportion of the genome of strain 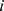 having ancestry in the corresponding populations, respectively. Let 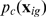 be the joint probability of the data from the 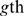 region for strain 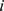 under population 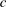. Then, the admixture model likelihood for the data in 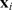 is determined by
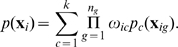
(6)The marginal posterior mode estimates of 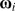 are obtained by numerical maximization combined with a simulation, to account for the uncertainty about 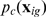 given the partition estimate 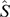. As illustrated by [14], the posterior distribution of 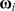 does not entirely plausibly represent the statistical uncertainty about 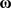, as the strain coefficients 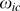 may in some cases have a mode in the interval from ∼.1 to ∼.2, while still reflecting only random fluctuations in 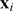 in the populations, in contrast to real ancestry in a particular population, say 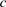. The issue was solved in [14] by calculating simulation-based 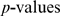 for 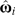 under the null hypothesis of no admixed ancestry. In the sequel, let 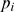 denote such a 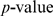 for an strain 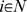.

We now combine the statistical tools from [14], and [15] to obtain an estimate 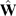 of 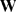. Firstly, populations are estimated using the posterior mode partition 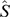. Then, for each identified population 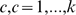, the extent of admixture events is estimated for 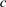 using a significance level 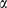, such that
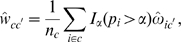
(7)where 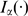 is an indicator function equal to one when the argument is true, and zero otherwise. The estimate (7) is for the population 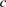 an average relative amount of (significant) DNA acquired from population 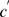, thus representing a combination of an average recombination intensity and the propensity of recombination events taking place between these populations. It should be noticed that the admixture model ignores possible contiguity of genes or genome regions. However, the genes present in MLST analyses tend to represent quite distant genome regions, which motivates the assumption of independence. If the genes are taken from a more contiguous region, it is possible to treat them as a single linked region in the model by concatenating the sequences prior to the genetic mixture analysis. Notice also that the above gene flow estimates can be complemented by investigating separately the rate and size of the exchange events. The rate of exchange events is represented by the proportion of strains in a population showing significant admixture from a particular source. In turn, the size of the exchange events is revealed by characterizing the values of the corresponding estimated admixture coefficients.

The latest version of BAPS (5.2) contains an implementation of the estimation procedure leading to (7), such that high-resolution images of the directed graph 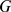 with the associated weight matrix 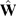 can be produced directly with the software. As illustrated in the RESULTS section, this facilitates the analysis of large data sets for which the numerical estimates of admixture can be very tedious to examine.

### Genetic shapes of the populations

The above presented statistical models and tools provide means for assessing the number of genetically isolated populations and the extent of recombination among them. However, this leaves open questions related to the underlying genetic population structure. In particular, the model summary estimates do not provide any information on the area occupied by the population in sequence space, which we term its genetic shape. By a genetic shape we refer both to the molecular heterogeneity present in a population, as well as the genetic affinities of its members towards other identified populations. We will illustrate that an investigation of the genetic shapes in this sense can yield useful characterizations of the population, pinpoint interesting subgroups of strains, and eventually provide clues to relate the genetic structure to some auxiliary information.

Let 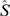 be the estimated genetic population structure and 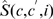 the structure where the strain 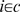 has been moved to the population 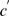. The relative genetic affinity of the strain 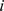 towards population 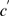 can be quantified in terms of the change in the log-predictive likelihoods
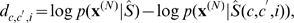
(8)which is always non-negative given that we have identified the true posterior optimum (4). However, even when 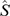 does not equal the global posterior optimum, our estimation algorithm is designed in such a way that negative values of (8) cannot be obtained, as any parameter configurations in the neighborhood of 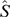 leading to an improvement of the posterior probability will be detected.

The difference (8) can be interpreted as the amount of information we lose in the prediction of the molecular characteristics in 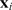 when the strain is assigned into another population, given that the remaining population structure is kept fixed. Thus, at the boundary, when (8) is equal to zero, no information will be lost. From the genetics perspective, the distribution of the values 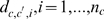, reflects the genetic shape of the population 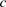 towards the population 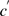. It is clear that this shape does not necessarily have an easily interpretable geometric configuration in low enough dimensions (1–3), such that it can be visualized. However, the shape of the distribution of 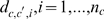, can still be used to reveal patterns of interest, which is illustrated in the RESULTS section.

To numerically characterize the genetic shape of a population using the values of (8) for 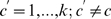, we use a kernel density estimate of the underlying distribution of the affinity measures. This is implemented in BAPS 5.2, which outputs graphical displays of the density curves. These are based on the standard Gaussian kernel with the Gaussian optimal bandwidth 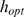 (see, e.g. [21]) according to
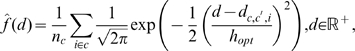
(9)where 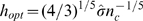, and further 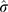 is the maximum likelihood estimate of the standard deviation of 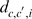 values. Such density curves will provide useful information concerning both the within and between population molecular variation as well as affinity.

### Simulated data

The simulated MLST data sets were generated by assuming a tentative gene flow graph 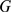 with the weight matrix 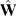 is changing randomly. The gene flow graph estimated by BAPS 5.2, denoted as 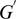, was then compared with 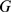 for evaluating the prediction accuracy. The characteristic of genetic shapes for the identified populations was also investigated for a wide range of scenarios given by 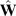.

**Figure 1:**
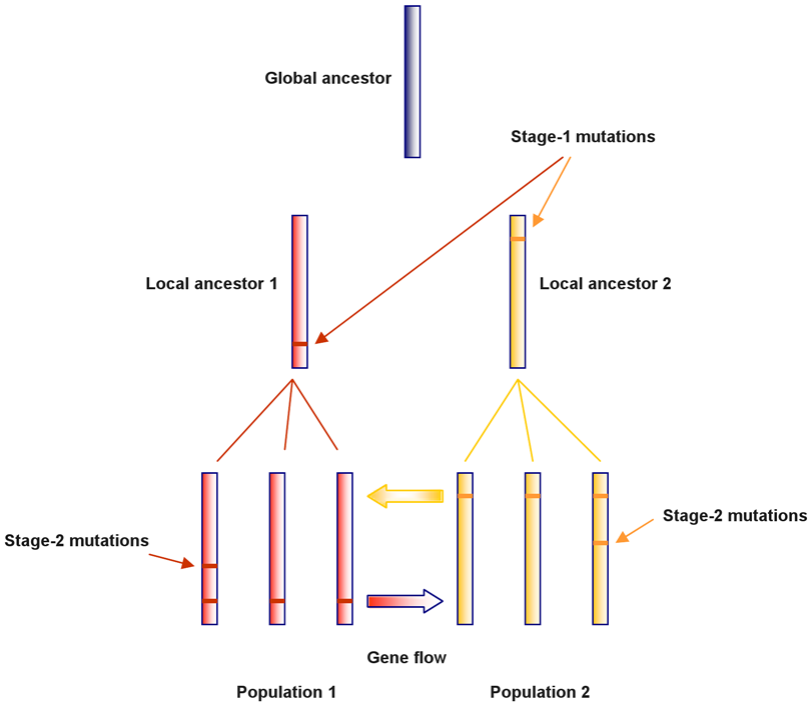
Graphical representation of the evolutionary model for a sample of two bacterial populations. Strain sequences are represented as vertical bars with horizontal lines indicating the mutations that have occurred since the global ancestor. Stage-1 mutations are defined as those that occurred on local ancestors which provide candidate sites for gene flow between the populations. Mutations that occurred after the local ancestors are referred to as stage-2 mutations.

The assumptions for the data generation are based on a simplified, yet reasonable evolutionary model of bacterial populations. We assumed that each population has a common local ancestor, and further back in time these local ancestors originated from a common ancestor of the whole population, termed as a global ancestor. This assumption enables a tree representation of the evolutionary relationships among the populations (Figure 1). It is important to note that we do not explicitly model the time at which these ancestral events occurred and therefore the edges in Figure 1 are in arbitrary length.

When ignoring recombination, the strains in a population will differ from each other only through the accumulation of point mutations. The mutations may have accumulated in two consecutive stages. In what follows, we referred to a mutation that occurred prior to the local ancestors as a *stage-1 mutation*, and a mutation that occurred afterwards as a *stage-2 mutation*. We assumed the infinite-site model of mutation, which implies that at most one mutation per site can occur in the DNA sequences [22]. This would imply that stage-1 mutations provide heterogeneity that leads to population diversification, while stage-2 mutations generate variations within a population. We further assumed that these two types of mutations occur independently of each other and result in a number of segregating sites. Let 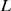 denote the total sequence length, then the expected number of segregating sites 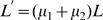, where 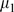 and 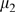 are the mutation rates for the two stages.

To simplify the problem, we considered recombinations that always lead to changes of DNA, so that they can be detected as admixture events. This corresponds to assuming that the DNA introduced by admixture needs to be distinctive compared to the homologous sites that have been observed within the population. Given the tree representation of the population evolution, such recombinations are restricted to occur at stage-1 mutation sites only.

Following these assumptions described above, the expected numbers of stage-1 and stage-2 mutation sites can be obtained by
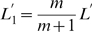
and
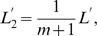
where 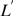 is the expected number of segregating sites; 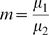 is the ratio of the two mutation rates. We considered the time length in stage-1 is longer than that in stage-2, so we did set 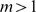. For data simulation we used 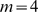 and 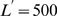, and set an equal population size 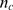 for all the populations. A simulated population data set thus contains 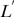 sites and 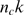 strains, where 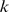 is the number of populations.

**Figure 2:**
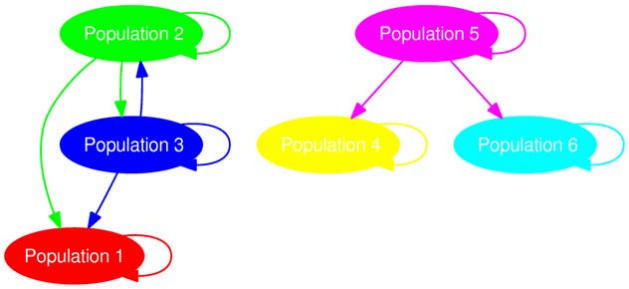
Tentative gene flow graph in six populations. The graph topology can be succinctly termed as 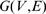, where the node set 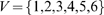 and the arrow set 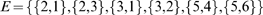. The actual rates of admixture associated with the arrows were randomly generated from a uniform distribution. Note the two ways of gene flow between population 2 and 3.

We specified a putative gene flow graph 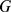 that consists of 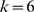 populations and the arrow set is specified in Figure 2. The rates of admixture between populations are characterized in the matrix 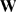, which is by definition a product of 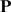 and 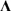. Therefore by simulating 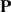 and 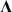 we can generate a parameter set in 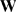 that conforms to the graph structure in Figure 2. We chose a consistent sampling scheme for 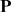 such that the diagonal elements 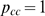 for 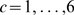, and the non-diagonal elements 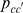 are uniformly distributed. 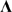 is also sampled from the Uniform distribution 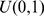, but with the row constraints 
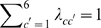
, since 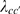 refers to the fraction of DNA sequence acquired from a particular source population.

Sampling a data set according to the putative population structure consists of three steps. First, a global ancestor of 500 segregating nucleotides was randomly simulated and for each of the six population a local ancestor was generated by randomly altering each nucleotide of the global ancestor with the probability 0.8. The sample strains for each population were generated by randomly mutating the local ancestor with the probability 0.2. The strains that have been recombined were randomly selected according to the parameter 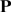, and for each of the recombined strain the actual amount of recombinations was determined by 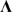.

Using the procedure described above, a population data set can be simulated for each of the selections of 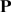 and 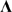. The population structure analysis was done with our Bayesian framework implemented in BAPS 5.2. We reported the accuracy of BAPS partition as choosing different values of 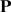 and 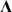. Once the true partition has been identified correctly, we assessed further the accuracy of the predicted gene flow structure, i.e. the similarity of graph topology between 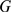 and 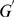. Note that the non-diagonal elements 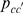 in 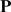 determine the propensity of acquiring DNA through recombination from 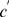 to 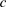, therefore a larger 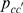 implies that the recombination would affect a higher proportion of strains in 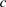. The increasing admixture propensity would make the recombination unidentifiable, since our Bayesian framework tends to favor the alternative hypothesis that the allelic frequency at the recombination site is more likely an effect of the stage-2 mutations, rather than a consequence of admixture. We therefore expected a negative correlation between 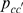 and the gene flow structure accuracy. We also expected that in order to obtain a reliable partition estimate, the non-diagonal elements 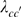 in 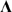 should be near zero, since a small rate of recombination along the DNA sequences might not perturb the population structure in a large scale. A large 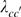, however, implies frequent recombination that might blur the population boundaries, so that the original population structure could be no longer identified.

### Real data

To illustrate the presented methods with a real data set, we applied BAPS 5.2 to MLST bacterial data. MLST(multi-locus sequence typing) is an approach to the unambiguous characterization of bacterial strains. The internal sequence of seven housekeeping genes, which include the *abc* Z, *adk*, *aro* E, *fum* C, *gdh*, *pdh* C and *pgm* genes are obtained and unambiguously characterize the strain. The strain sequences are generally reported to the publicly accessible MLST strain databases (see, e.g. http://www.mlst.net), which are currently hosting a fast growing number of bacterial genera and also a few eukaryotic organisms.

We chose the *Neisseria* species for validation as homologous recombination is known to be frequent in both *N. meningitidis* and *N. lactamica* species [23],[24]. Furthermore, occasional horizontal gene flow over the species boundary has also been observed [25]. However, it is unclear to what extent the gene flow occurs and its consequence for population structure. To investigate this we applied BAPS 5.2 to a MLST sample which contains 4823 strains of *N. meningitidis* and 263 strains of *N. lactamica*. The data was accessed for analysis from the *Neisseria* MLST database at 17/3/2006 [26].

For such MLST type sequence data, we utilized the genetic linkage model [15] to account for dependency within the neighboring nucleotide bases. To assess the ability of our methods to find correctly or nearly correctly the populations hiding in the data, we considered a simulation scenario for generating a bootstrap sample which contains the strains randomly selected from a sub-collection of the identified *Neisseria* populations, using the procedure as follows:

1. Decide the number of clusters 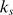 in the bootstrap data. We consider five scenarios where in each scenario 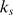 is one of 5,10,15,20 and 25.
2. Select randomly 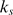 clusters without replacement from the identified population structure based on the complete data.
3. For each chosen cluster, sample with replacement a random number of strains. The number of sampled strains follows the uniform distribution in the range of (30, 80).
4. Clustering the data generated in step 3 using BAPS 5.2.

By repeating the scenario multiple times (we use 5 repeats) for each 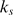, we can check how well the resulting partition agrees with the chosen 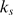 and how close the partition is to the general setting. This approach allows us to investigate the statistical power to correctly detect populations when the number of available strains is quite limited per population.

Conditional on the identified population structure, comparative rates of admixture between the populations can be further estimated and summarized in a gene flow graph. We plotted the genetic shapes of several populations in *N. meningitidis* which show significant gene flow towards the *N. lactamica* species, and also compared their similarity in terms of admixture tendency.

To investigate whether the signals of admixture varied considerably over the seven genes, we performed a bootstrap analysis where a single gene at a time was excluded when inferring the rates of admixture. The analysis was performed conditional on the clusters identified using the original complete data set. The relative importance of each gene could then be tentatively summarized by calculating for each cluster the average changes in incoming and outgoing gene flow while treating the estimates from the complete data as a baseline.

Phylogenetic analysis of the *Neisseria* data was performed using MEGA v.4.0.2 [27]. Neighbor-Joining (NJ) tree was constructed with the maximum composite likelihood model assuming rate uniformity and pattern homogeneity. eBURST analysis of the *Neisseria* data was performed using the default options in the online version 3 available at http://eburst.mlst.net [28].

## Results

### Simulated data

**Figure 3:**
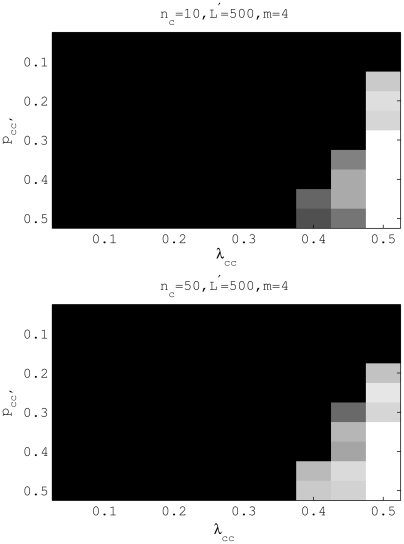
Testing partition accuracy for different choices of gene flow weights for a small population size 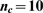 (upper panel) and a large population size 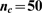 (lower panel). The number of segregating sites for both settings is 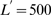 and the ratio of mutations at two stages is 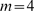. Data were generated by assigning 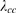 and 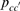 randomly at the interval [0,1] with the gene flow topology fixed as in Figure 2. A brighter area corresponds to a range of 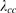 and 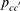, within which the true partition has been identified by BAPS with a higher accuracy as measured by Rand Index (RI).

We reported the partition accuracy with respect to different choices of 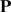 and 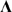 under a constant population size 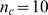 in one scenario and 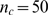 in another. The partition accuracy measured by the Rand Index (RI) (see e.g. [29]) is summarized as a grey-scale map (Figure 3). In the presence of a small amount of admixture, *i*.e. 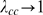, the tentative population structure can be identified with high accuracy. As the recombination rate increases over a critical threshold, *e*.g. as 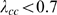 for the current setting, the partition accuracy drops quickly. Therefore, a higher recombination rate, indicated by a lower 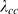, would imply a lower partition stability. Such an observation matches our expectation that excessive amount of admixture tends to obscure the putative population structure.

**Figure 4:**
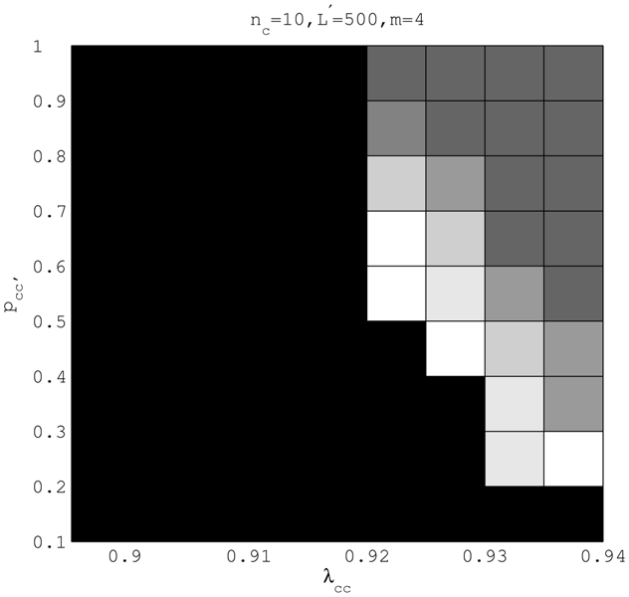
Testing gene flow structure accuracy for 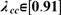 and 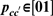. Graph similarity was measured in the Hamming distance coded in a gray-scale image. Cells with the paper white color represent the scenarios where the partition and the gene flow structure in Figure 2 are both correctly identified by BAPS.

We may look further into the gene flow graph prediction only if the genetic structure (i.e. the true partition) is correctly identified. We used Hamming distance as a measure of gene flow structure accuracy and the result is shown in Figure 4. The gene flow graph structure can be satisfactorily discovered when 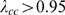 and 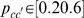. However, a negative correlation between 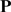 and 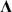 was also noticeable. This result suggests that if admixture affects a population through a small proportion of strains, then the chances of its correct estimation by BAPS 5.2 are high. In contrast, admixture that occurred at most of the strains is more likely to be ascribed to variation arising within the population by mutation. These observations are in harmony with the investigation of the effect recombination intensity on the emergence of distinct populations for a bacterial species in [3]. Extensive levels of recombination will act as a cohesive force keeping populations together as a large gene pool, which consequently prevents the statistical detection of the recombination in terms of such a population genetic model as investigated here. This is entirely reasonable, because any substantial genetic population boundaries will not exist under such circumstances, and consequently, recombinations over population boundaries are not meaningfully defined, let alone detectable by a statistical model. Moreover, if certain parts of the data are too weak for reliable admixture inferences due to very small population cardinalities in the genetic mixture estimate, it is possible to leave the admixture coefficients undetermined for them using the option available in BAPS, as discussed in [14]. The extensive simulation study performed by [30], showed that the BAPS inferences about the genetic structure were generally sensible from a phylogenetic perspective, even in the presence recombination events, provided that the data are at least reasonably informative. With very weakly informative molecular data, it cannot be expected that any detailed statistical population genetic model would provide highly accurate estimates of the population characteristics.

**Figure 5:**
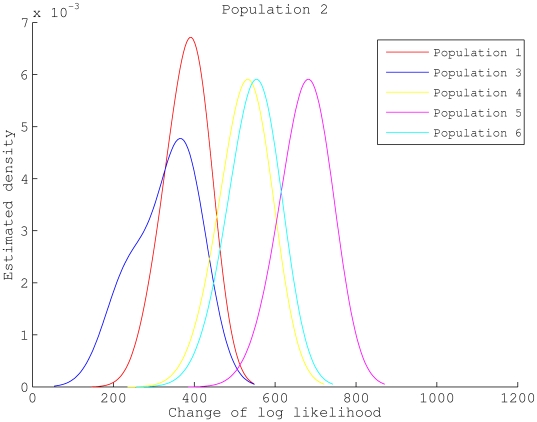
Genetic shapes of five populations relative to population 2. The data set was generated with 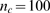, 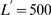, 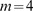 and Figure 2 as the underlying population structure. Each curve is a density estimation of (8) using (9) for one target population.

We used a simulated data set for illustration of genetic shapes represented as the density estimation in (8). The data set was generated with 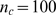 and 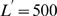. Figure 5 shows the estimated genetic shapes using population 2 as the reference, as compared to the other five populations. It can be seen from Figure 5 that the influence of admixture between the populations is reflected also on the genetic shapes. For example, the density curves for population 1 (red) and for population 3 (blue) are more shifted towards zero than the other populations, and hence imply a closer relationship to population 2. This is not surprising since population 2 is a common donor of DNA to populations 1 and 3 (Figure 2). On the other hand, the density curve for population 3 appears to have two modes, which is a feature exhibited in neither population 1 nor any other populations. Note that population 3 is the only population which donates DNA to population 2. We might use the bi-modality of a density curve as a potential indicator of gene flow to the reference population.

### The *Neisseria* data

**Figure 6:**
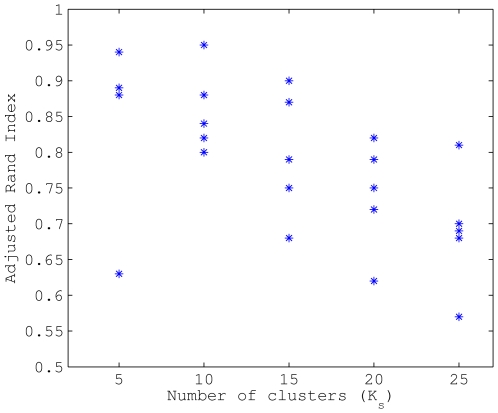
Bootstrap mixture analyses of the *Neisseria* data. The figure shows the adjusted rand index between the partition based on the original data and the alternative based on a bootstrap data set by resampling in 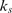 clusters. Five repetitions were made for each of the 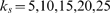 clusters.

In total 32 BAPS populations are identified, where three populations (numbered as 8, 29 and 32) belong to the *N. lactamica* species and the remaining 29 populations are labeled as *N. meningitidis* species. For accessing the robustness of the identified population structure, the partition determined using the whole data set was compared with the partition using bootstrap data generated according to the simulation scenarios. Figure 6 shows the adjusted Rand Index as a result of the comparison. Our partition method is able to identify the population structure with good accuracy, even though the performance may decrease as the complexity level of the data increases and when the number of available strains per population is quite low. It should be noted that the number of strains in the bootstrap samples was typically much smaller than the number of strains assigned to a particular population in the analysis of the original data. This illustrates that the population identification becomes highly stable when the sample sizes are sufficiently large.

**Figure 7:**
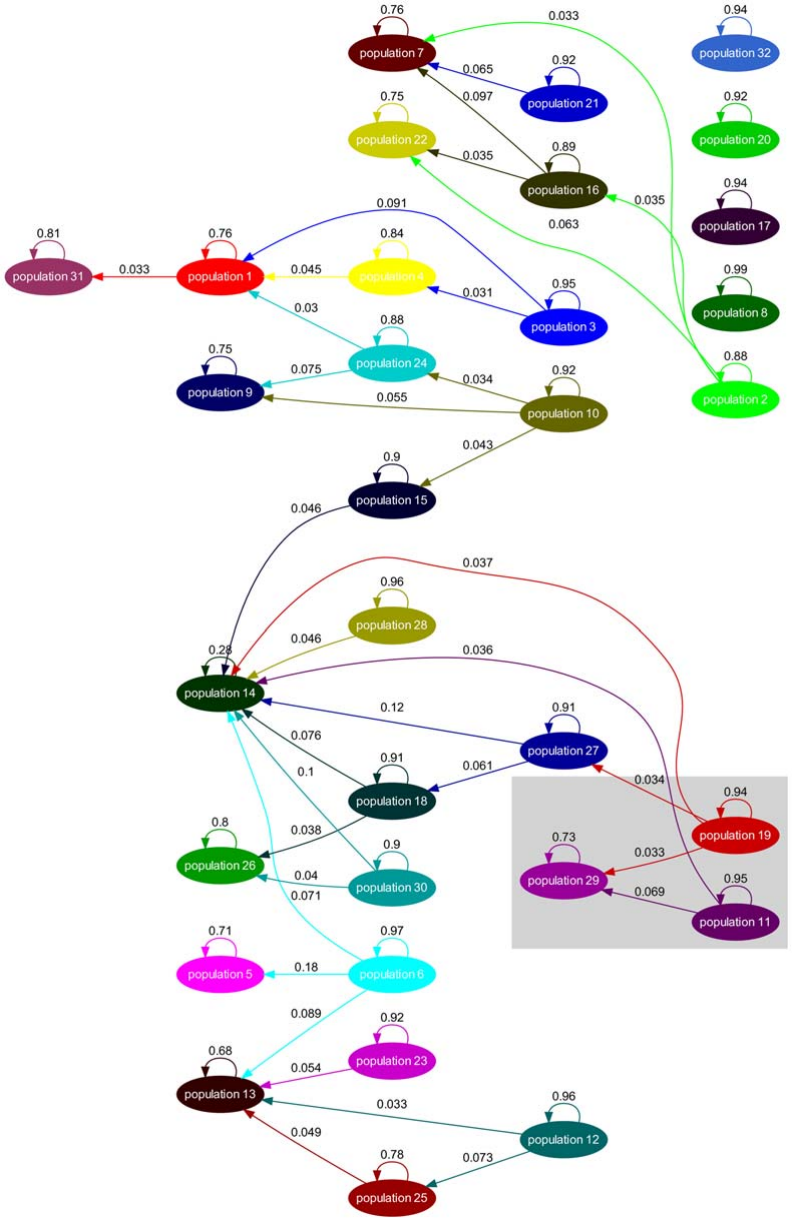
Gene flow network identified in the *N. meningitidis* and *N. lactamica* populations. To investigate the ancestral admixture of a certain population, one can look at all the arrows pointing at this population. A typical population contains the major sources of its own, denoted as a self-looping arrows, and small proportions of gene flow from other populations. For instance, population 29 has 73% of its own genetic makeup and 27% of the DNA introduced via gene flow from other populations. Two major sources of gene flow for population 29 are population 19 and population 11, with 3.3% and 6.9% in the contribution separately. The remaining 17.1% of genes comes from various sources while none of them contributes a proportion larger than 3% and therefore are not displayed due to the pruning.

The results of admixture analysis for the *Neisseria* data set are summarized in Figure 7. The graph was obtained by fixing the admixture significance threshold 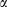 at 0.05 and then pruning the arrows with gene flow strength below 0.03. It can be seen from the grey box highlighted in Figure 7 that two admixture arrows that imply inter-species gene flow remain significant, where two of the *N. meningitidis* populations (11 and 19) are constantly influencing the genetic makeup of one population of *N. lactamica* (population 29). The admixture arrows are uniformly directed from *N. meningitidis* towards *N. lactamica*, implying that *N. meningitidis* might donate genetic materials into *N. lactamica*, while the gene flow in a reversed direction is not supported by the analysis.

The admixture estimates for the 32 clusters obtained under the bootstrap analysis over the genes are summarized in Table 1. To see how much the exclusion of a particular gene changes the estimates, we may look at the overall consistency of the inferred average outgoing and incoming gene flow using the complete data case as reference. It is observed that the exclusion of gene *gdh* seems to affect the admixture consistency most, as in this case the changes of outgoing (incoming) gene flow reach the maximum value in 20 (13) of the 32 clusters. In contrast none of the clusters is experiencing the largest gene flow changes when either the gene *adk* or *aroE* is excluded, suggesting that recombination signals on these two genes are more marginal.

**Table 1:** Bootstrap admixture analyses of the *Neisseria* data.

	Complete	abcZ_excluded	adk_excluded	aroE_excluded	fumC_excluded	gdh_excluded	pdhC_excluded	pgm_excluded
Cluster 1	0.0030(0.0102)	0.0060(0.0137)	0.0030(0.0102)	0.0030(0.0101)	0.0026(0.0094)	0.0068(0.0195)	0.0108^*^(0.0097)	0.0034(0.0196^†^)
Cluster 2	0.0061(0.0062)	0.0079(0.0047)	0.0056(0.0065)	0.0063(0.0063)	0.0057(0.0061)	0.0125^*^(0.0172)^†^	0.0103(0.0056)	0.0105(0.0112)
Cluster 3	0.0119(0.0015)	0.0110(0.0012)	0.0119(0.0016)	0.0126(0.0017)	0.0124(0.0016)	0.0155^*^(0.0018)	0.0129(0.0016)	0.0122(0.0022^†^)
Cluster 4	0.0048(0.0082)	0.0102(0.0101)	0.0051(0.0085)	0.0043(0.0089)	0.0047(0.0085)	0.0078(0.0204^†^)	0.0126(0.0070)	0.0146^*^(0.0073)
Cluster 5	0.0016(0.0131)	0.0040(0.0217)	0.0016(0.0117)	0.0014(0.0129)	0.0017(0.0119)	0.0042^*^(0.0243^†^)	0.0041(0.0099)	0.0039(0.0124)
Cluster 6	0.0210(0.0017)	0.0460^*^(0.0020)	0.0199(0.0017)	0.0202(0.0015)	0.0188(0.0016)	0.0359(0.0027)	0.0222(0.0017)	0.0197(0.0035^†^)
Cluster 7	0.0042(0.0097)	0.0053(0.0094)	0.0041(0.0095)	0.0041(0.0095)	0.0042(0.0090)	0.0122^*^(0.0171^†^)	0.0060(0.0086)	0.0073(0.0138)
Cluster 8	0.0010(0.0004)	0.0008(0.0005)	0.0008(0.0003)	0.0009(0.0005)	0.0153^*^(0.0012^†^)	0.0012(0.0007)	0.0009(0.0005)	0.0009(0.0003)
Cluster 9	0.0039(0.0092)	0.0032(0.0208^†^)	0.0040(0.0089)	0.0036(0.0090)	0.0038(0.0086)	0.0072^*^(0.0136)	0.0056(0.0073)	0.0049(0.0060)
Cluster 10	0.0097(0.0045)	0.0126(0.0099^†^)	0.0102(0.0042)	0.0094(0.0047)	0.0107(0.0041)	0.0182^*^(0.0074)	0.0139(0.0040)	0.0163(0.0063)
Cluster 11	0.0075(0.0019)	0.0066(0.0015)	0.0078(0.0018)	0.0082(0.0018)	0.0074(0.0020)	0.0143^*^(0.0031)	0.0071(0.0014)	0.0097(0.0057^†^)
Cluster 12	0.0104(0.0013)	0.0146(0.0025^†^)	0.0098(0.0012)	0.0103(0.0014)	0.0104(0.0013)	0.0156^*^(0.0019)	0.0109(0.0008)	0.0081(0.0012)
Cluster 13	0.0016(0.0108)	0.0015(0.0068)	0.0012(0.0105)	0.0014(0.0102)	0.0015(0.0107)	0.0045^*^(0.0180)	0.0020(0.0221^†^)	0.0013(0.0097)
Cluster 14	0.0002(0.0246)	0.0002(0.0219)	0.0002(0.0248)	0.0001(0.0252)	0.0002(0.0255)	0.0002(0.0301^†^)	0.0010^*^(0.0247)	0.0002(0.0234)
Cluster 15	0.0102(0.0034)	0.0125(0.0021^†^)	0.0101(0.0036)	0.0112(0.0045)	0.0093(0.0043)	0.0131^*^(0.0053)	0.0082(0.0053)	0.0092(0.0031)
Cluster 16	0.0108(0.0040)	0.0115(0.0070)	0.0107(0.0042)	0.0109(0.0044)	0.0099(0.0047)	0.0174(0.0069)	0.0116(0.0038)	0.0100(0.0035)
Cluster 17	0.0018(0.0019)	0.0075^*^(0.0077^†^)	0.0018(0.0019)	0.0019(0.0015)	0.0015(0.0016)	0.0030(0.0013)	0.0017(0.0017)	0.0019(0.0011)
Cluster 18	0.0069(0.0029)	0.0063(0.0053^†^)	0.0077(0.0028)	0.0071(0.0029)	0.0066(0.0033)	0.0114^*^(0.0051)	0.0095(0.0027)	0.0083(0.0022)
Cluster 19	0.0090(0.0022)	0.0098(0.0024)	0.0090(0.0021)	0.0093(0.0024)	0.0099(0.0025)	0.0115(0.0033^†^)	0.0063^*^(0.0021)	0.0087(0.0022)
Cluster 20	0.0014(0.0036)	0.0021(0.0023)	0.0013(0.0034)	0.0016(0.0035)	0.0016(0.0035)	0.0024(0.0180^†^)	0.0016(0.0029)	0.0026^*^(0.0031)
Cluster 21	0.0067(0.0031)	0.0068(0.0028)	0.0065(0.0032)	0.0069(0.0029)	0.0083(0.0029)	0.0111^*^(0.0057^†^)	0.0059(0.0031)	0.0070(0.0054)
Cluster 22	0.0025(0.0068)	0.0025(0.0049)	0.0022(0.0068)	0.0023(0.0066)	0.0023(0.0065)	0.0182^*^(0.0069)	0.0003(0.0313^†^)	0.0025(0.0061)
Cluster 23	0.0064(0.0044)	0.0105(0.0221^†^)	0.0062(0.0048)	0.0064(0.0044)	0.0061(0.0041)	0.0170^*^(0.0081)	0.0090(0.0043)	0.0059(0.0031)
Cluster 24	0.0075(0.0052)	0.0142(0.0072)	0.0079(0.0049)	0.0078(0.0049)	0.0058(0.0056)	0.0152^*^(0.0069)	0.0080(0.0061)	0.0081(0.0137^†^)
Cluster 25	0.0043(0.0095)	0.0016^*^(0.0252^†^)	0.0043(0.0091)	0.0046(0.0101)	0.0045(0.0089)	0.0065(0.0172)	0.0065(0.0093)	0.0048(0.0073)
Cluster 26	0.0008(0.0095)	0.0011(0.0078)	0.0007(0.0102)	0.0007(0.0097)	0.0005(0.0089)	0.0020(0.0200^†^)	0.0028^*^(0.0170)	0.0014(0.0094)
Cluster 27	0.0083(0.0039)	0.0103(0.0055)	0.0080(0.0041)	0.0083(0.0039)	0.0087(0.0041)	0.0146^*^(0.0101^†^)	0.0119(0.0037)	0.0083(0.0039)
Cluster 28	0.0050(0.0015)	0.0052(0.0020)	0.0049(0.0014)	0.0051(0.0015)	0.0055(0.0016)	0.0076^*^(0.0021^†^)	0.0052(0.0012)	0.0050(0.0018)
Cluster 29	0.0007(0.0054)	0.0011(0.0066)	0.0012(0.0050)	0.0011(0.0060)	0.0013^*^(0.0048)	0.0011(0.0068)	0.0012(0.0059)	0.0005(0.0079^†^)
Cluster 30	0.0054(0.0047)	0.0062(0.0032)	0.0050(0.0048)	0.0055(0.0048)	0.0052(0.0047)	0.0069(0.0179^†^)	0.0063(0.0037)	0.0079^*^(0.0087)
Cluster 31	0.0071(0.0058)	0.0075(0.0046)	0.0071(0.0057)	0.0069(0.0057)	0.0067(0.0058)	0.0103^*^(0.0064)	0.0072(0.0137^†^)	0.0070(0.0054)
Cluster 32	0.0016(0.0022)	0.0013(0.0025)	0.0019(0.0020)	0.0017(0.0022)	0.0013(0.0153^†^)	0.0027^*^(0.0025)	0.0010(0.0019)	0.0018(0.0032)

For each cluster the average outgoing (incoming) gene flow is shown for each set of genes used in the analyses. The baseline estimates using all of the genes are listed in the Complete column. The average outgoing gene flow for a cluster 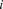 is the mean value of 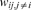, where 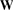 is the estimated gene flow matrix using the significance threshold 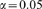. The average incoming gene flow is obtained as the mean of 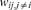. For each cluster the gene set associated with the largest change in gene flow estimates is highlighted for both outgoing and incoming arrows, marked with * and †, respectively.

**Figure 8:**
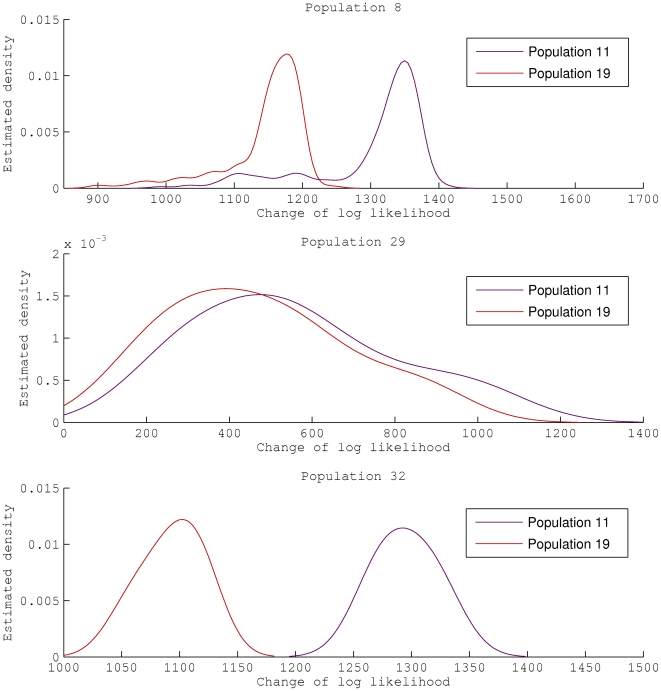
Genetic shapes of *N*. lactamica populations 8, 29 and 32 as relative to *N*. meningitidis populations 11 and 19.

We plotted in Figure 8 the estimated densities of 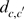 for the three *N. lactamica* populations (8, 29, 32), relative to *N. meningitidis* populations 11 and 19 separately. The densities for population 29 have a tendency towards zero, suggesting a close genetic affinity with populations 11 and 19. In contrast, the densities of populations 8 and 32 are much further away from zero, implying a distinctive difference in their genetic makeup compared to *N. meningitidis* populations 11 and 19. This result is consistent with the admixture pattern presented in Figure 7.

The eBURST analysis of the *Neisseria* database resulted in 253 groups and 1165 singleton strains. The biggest group consists of 795 strains and there are additionally four groups containing more than 200 strains. Table 2 shows the degree of concordance between the eBURST groups and BAPS populations. Due to the very large number of eBURST groups, only groups containing at least 20 strains were included in this comparison. Table 2 shows that the largest eBURST groups harbour many strains from multiple BAPS populations, whereas the vast majority of strains in smaller groups are typically found only in a single BAPS population (in some cases in two populations). As these cases represent single-locus variants of one another from eBURST analysis being clustered into different populations by BAPS, it means that there must exist a very large amount of anomalous variation at the nucleotide level within the other locus to allow the model to identify such subgroups. It should also be kept in mind that the BAPS model used for the identification of these populations is not a phylogenetic method in contrast to eBURST, which is an important distinction particularly in the presence of highly recombinogenic data. Out of the three BAPS populations of *N. lactamica* strains (populations 8, 29 and 32), only one forms a group in the eBURST analysis (group 14, Table 2). Strains in the other populations are primarily assigned to singleton groups. This difference is further explored below using a phylogenetic analysis at the nucleotide level.

**Table 2:** The number of strains that are jointly assigned into a BAPS population and a EBURST group.

		EBURST groups																										
		1	2	3	4	5	6	7	8	9	10	11	12	13	14	15	16	17	18	19	20	21	22	23	24	25	26	27
BAPS populations	1	4	1	17	1	3	1	0	0	0	1	0	0	0	0	2	0	1	1	2	0	0	0	3	2	0	0	0
	2	14	7	0	0	2	3	4	91	0	1	3	0	1	0	5	0	0	0	0	0	0	0	0	0	2	0	0
	3	0	0	141	0	0	0	0	0	0	0	0	0	0	0	0	0	0	0	0	0	0	0	0	0	0	0	0
	4	48	9	3	2	0	0	1	3	2	0	0	0	0	0	0	0	2	0	27	0	0	0	0	0	2	0	0
	5	72	12	4	0	0	0	0	5	0	6	1	2	0	0	0	0	0	0	0	0	0	0	0	0	0	0	0
	6	274	1	0	0	0	0	0	0	0	0	0	0	0	0	0	0	0	0	0	0	0	0	0	0	0	0	0
	7	1	1	4	0	0	11	0	6	0	4	0	0	0	1	25	0	0	0	0	3	0	0	0	16	0	0	0
	8	0	0	0	0	0	0	0	0	0	0	0	0	0	0	0	0	0	0	0	0	0	0	0	0	0	0	20
	9	1	0	0	1	0	0	0	0	0	0	0	0	0	1	0	0	0	0	0	1	0	0	1	0	0	0	0
	10	5	0	0	132	2	0	7	0	11	0	0	0	0	0	0	1	0	0	0	0	0	0	0	2	0	0	0
	11	0	140	0	0	0	0	0	0	0	0	0	0	0	0	0	0	0	0	0	0	0	0	0	0	0	0	0
	12	0	0	0	0	0	0	0	0	0	69	0	0	0	0	0	0	0	0	0	2	0	0	0	0	0	0	0
	13	39	1	0	0	0	0	0	0	0	0	0	0	0	0	0	0	0	0	0	0	1	1	0	0	0	18	0
	14	37	7	3	3	16	11	0	0	0	1	2	0	1	0	0	0	1	3	0	1	2	1	0	1	1	0	0
	15	0	0	0	0	174	0	0	0	0	0	0	0	0	0	0	0	0	0	0	0	0	0	0	0	0	0	0
	16	0	0	0	3	2	89	0	0	0	0	0	0	0	0	0	0	0	0	0	0	0	0	0	0	0	0	0
	17	38	0	0	0	0	0	0	0	0	0	0	0	0	0	0	0	0	0	0	0	0	0	0	0	0	0	0
	18	0	0	2	0	0	0	0	0	0	0	0	2	47	0	1	0	0	0	0	0	0	0	0	0	0	0	0
	19	0	0	0	0	0	0	106	0	0	0	0	0	0	0	0	0	0	0	0	0	0	0	0	0	0	0	0
	20	0	3	0	0	2	0	0	1	0	0	0	0	0	0	0	30	0	0	0	22	0	0	0	0	0	0	0
	21	0	0	0	1	0	0	0	0	0	0	59	0	0	0	0	2	0	2	0	0	0	0	0	0	0	0	0
	22	2	0	3	0	3	0	0	2	2	0	1	0	0	0	0	0	0	0	2	0	1	2	0	0	0	0	0
	23	176	0	0	0	0	1	0	1	0	3	0	0	1	0	2	0	0	0	0	0	0	0	0	0	0	3	0
	24	0	0	0	77	1	5	1	0	11	0	0	0	0	0	0	0	0	26	0	0	0	0	2	2	0	0	0
	25	68	0	1	9	7	1	1	0	0	5	1	0	0	0	0	0	0	0	0	0	0	0	0	0	0	0	0
	26	1	3	2	0	0	0	0	0	3	0	0	2	0	0	0	0	0	0	0	0	21	11	0	0	0	0	0
	27	1	0	62	1	2	0	0	0	0	0	0	58	0	0	0	1	0	0	0	0	0	0	0	0	0	0	0
	28	0	0	0	0	0	0	0	0	0	0	0	0	0	46	0	0	0	0	0	0	0	0	0	0	0	0	0
	29	0	0	0	0	0	0	0	0	0	0	0	0	0	0	0	0	0	0	0	0	0	0	0	0	0	0	0
	30	13	96	2	0	1	0	0	1	0	1	0	0	1	0	0	0	29	0	0	1	0	0	0	0	18	0	0
	31	1	0	1	1	0	0	0	0	66	0	0	0	0	0	0	0	0	1	0	0	0	10	17	0	0	0	0
	32	0	0	0	0	0	0	0	0	0	0	0	0	0	0	0	0	0	0	0	0	0	0	0	0	0	0	0

Only the EBURST groups that contain more than 20 strains are listed.

**Figure 9:**
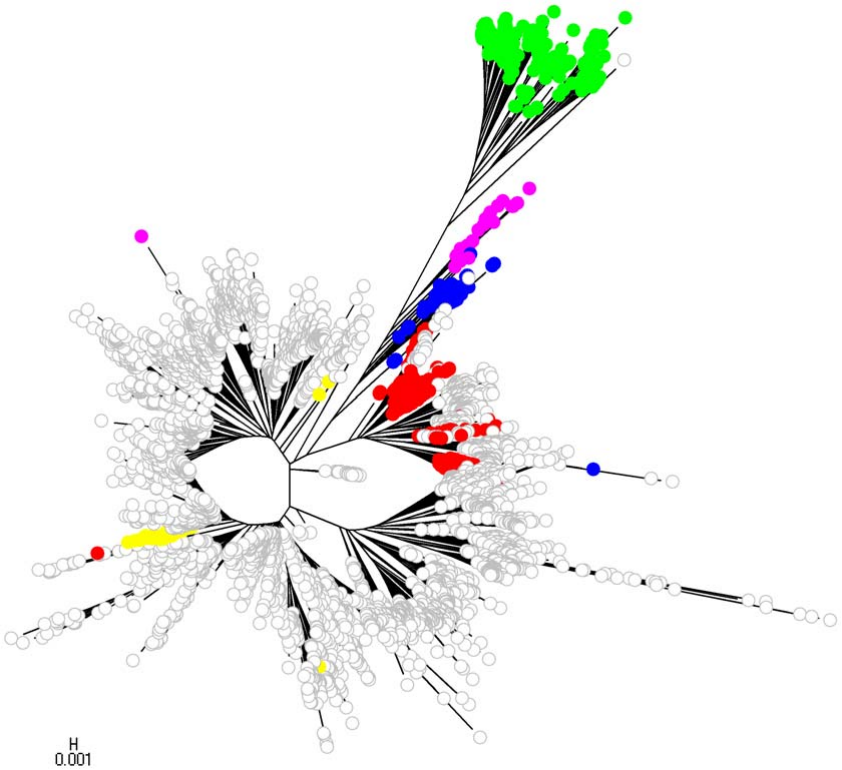
The first NJ tree. It shows a subset of the BAPS populations indicated with distinct colors.

To facilitate comparison of the phylogenetic analysis with the partition yielded by BAPS, we labelled strains with colors indicating population memberships. However, given the large number of strains included in the analysis and the large number of populations inferred by BAPS, it would be very challenging to visually extract information from a single NJ tree harbouring all the populations simultaneously. Therefore, four separate NJ trees are displayed in Figures 9–12, each of which shows a subset of the BAPS populations indicated with distinct colors. The strains remaining outside this particular subset are indicated by white circles. Since it is difficult to specify more than approximately 20 colors which remain clearly distinguishable from each other, independent coloring schemes were used for each tree to show the phylogenetic composition of the populations. Thus, it is not possible to compare the color codes directly with those in the gene flow network in Figure 7. The color coding scheme for the populations is shown in Figure 13 to enable comparison of the phylogenetic analysis and the gene flow network.

**Figure 10 pcbi-1000455-g010:**
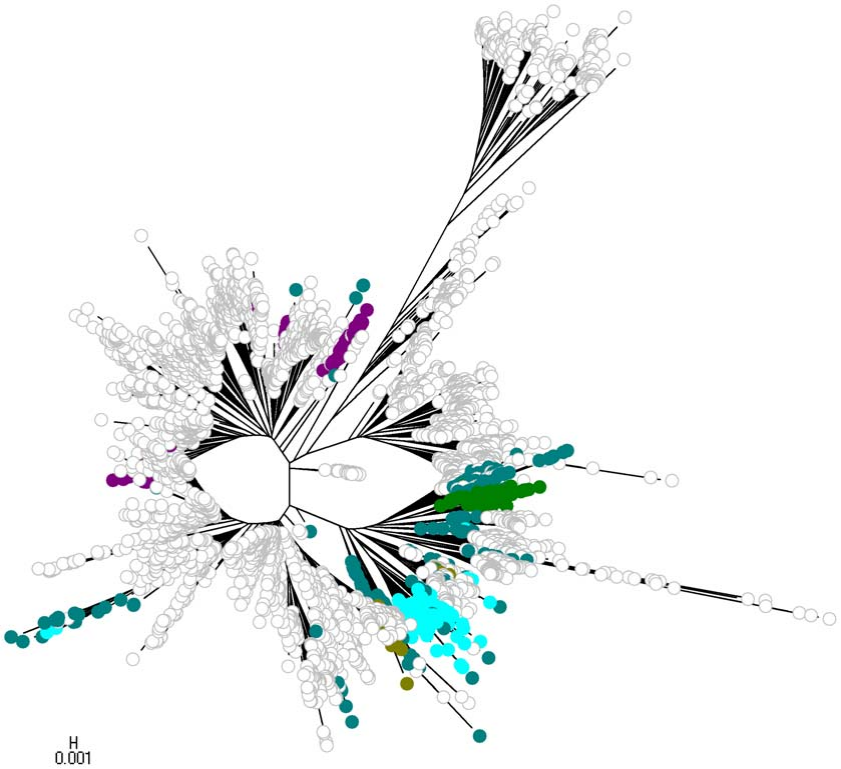
The second NJ tree.

**Figure 11 pcbi-1000455-g011:**
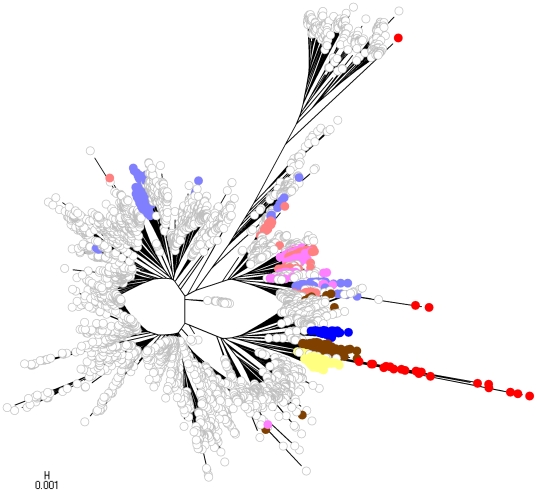
The third NJ tree.

**Figure 12:**
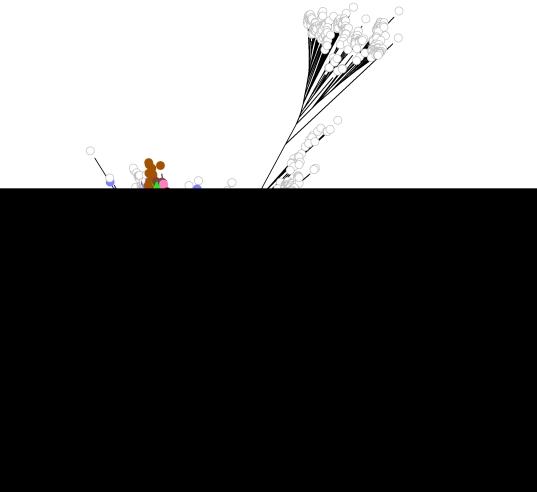
The fourth NJ tree.

**Figure 13:**
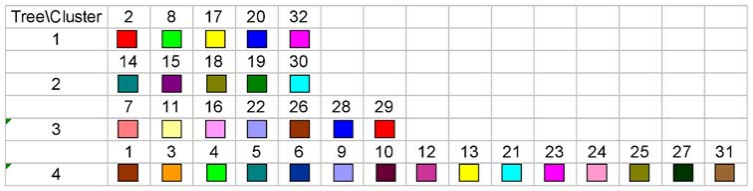
Color coding scheme used in the BAPS populations and the NJ trees. For example, the first row shows the BAPS populations highlighted in the first NJ tree in Figure 9.

The assignment of the populations to the NJ trees reveals that while a considerable number of them form relatively tight groups of lineages, there are also many populations in which the strains are spread over several separate lineages in the tree. This illustrates the dilution of phylogenetic signals in the presence of considerable levels of recombination between populations of strains. The population (population 14) which according to the inferred gene flow network is the most prominent recipient of genetic material from a multitude of sources, is seen (Figure 10) to include some dense and relatively large groups of strains that are found in separate parts of the tree. In addition, this population harbors a number of tiny groups of strains scattered over the three.

In the BAPS analysis, strains identified as *N. lactamica* fell into three populations: 8, 29 and 32. Figure 7 indicates that we found no evidence for significant admixture involving populations 8 and 32. Population 29 however was found to be associated with variation characteristic of populations 11 and 19, which were composed of meningococcal strains. The positions of the STs composing these five BAPS populations and one other (8, 29, 32, 19, 11 and 20) are shown in Figure 7. The isolated status of population 8 is apparent as a well resolved group, whereas the recombinant status of 29 is clear from the way these STs are scattered around the tree with long branch lengths originating apparently separately from the main *N. lactamica* population. The role of meningococcal strains in populations 11 and 19 in this is evident, in that the recombinant *N. lactamica* strains (population 29, shown in red) apparently originate close to these populations in the main meningococcal radiation.

Population 32 is intermediate on the tree between the majority of *N. lactamica* strains and the main meningococcal radiation. Hence these STs may be considered as examples of the so-called fuzzy fringes which have been proposed for recombinogenic species [25]. As noted however, they were not associated with significant admixture in the estimated gene flow network (Figure 7). Close examination of population 32 shows that 4 of the 22 STs in the population exhibited significant admixture with population 20 (shown in blue), receiving on average 12.3% from this population (which is composed of strains identified as meningococcus). It is interesting to note that populations 32 and 20 adjoin each other in the tree.

## Discussion

In the present work we have introduced statistical tools implemented in the BAPS software that enable analyses of bacterial population structures on a previously unprecented scale, as the computational complexity of the earlier standard Bayesian methods prevents their application to large databases associated with complex patterns of admixture. This is particularly important when at least a moderate number of recombination events have plausibly taken place in a population, as a statistically valid characterization of the population structure then requires fairly extensive sampling of strains. It was also noted that a standard MCMC-based approach is not expected to yield a viable strategy for such analyses in practice, due to both the time constraints as well as the statistical accuracy of the resulting estimates. The BAPS analysis (inference about the populations and the levels of admixture) of the *Neisseria* database was completed within roughly 95 CPU hours on a standard PC with a 2.8 GHz Pentium 4 processor. As a comparison, our initial experiments with the STRUCTURE software [8] suggest conservatively that a comparable analysis had taken at least several thousands of CPU hours on the same machine. Moreover, the convergence problems associated with the Gibbs sampler algorithm, when applied to mixture models (e.g. [10]), suggest that statistically reliable estimates of the population structure are likely not accessible for data sets of such a high degree of complexity.

The presented methods can be effectively utilized in a variety of contexts, where the genetic population structure is relevant, e.g. for the investigation of epidemiological questions and experimentally derived features of bacterial strains. For instance, outlying groups with specific characteristics with respect to virulence or antibiotic resistance may be detected from large population samples.

The concept of a genetic shape, which was introduced here to represent the molecular variability of an identified population and its affinity towards other populations as a whole, is an intriguing characteristic associated with considerable potential for further theoretical research. Namely, the average change of log predictive likelihoods between populations can also be interpreted as the change in ‘free energy’ associated with a gene flow event. The larger this quantity, the more likely the ‘reaction’ of gene flow could occur spontaneously. However, it is not trivial to determine the minimal energy level that triggers such events. From the analysis of simulated data (results not shown) we are expecting that such an energy threshold depends on the identified population sizes. In particular, the analogy with physics-based characterizations of molecular interaction systems could yield mathematical ways to predict horizontal transfer events.

Although this integrated approach advocated here provides a feasible means of handling data from thousands of strains and a multitude of genes, several issues remain. Firstly, if a very large number of genes are considered, it is likely that not all of them will be present or functional in overall in a heterogeneous population at a genus level. Under such circumstances it would be necessary to develop further the Bayesian model for a population structure and admixture to take into account that not all molecular information is shared by the sampled strains. Secondly, the scalability of the stochastic learning algorithms should be improved to ensure that models could still be fitted to data without access to supercomputing facilities. Given the present rate of improvement in sequencing facilities, it is likely that the need for such large-scale analyses will be a reality within a relatively short time-span. In order to meet these needs in the future, we are currently investigating several theoretical approaches to develop further the statistical population genetic tools available in BAPS.

The findings from the combined phylogenetic and population genetic analyses suggest possible events of convergence between *N. lactamica* and *N. meningitidis* that have arisen on multiple occasions and have occurred for clearly separate lineages of the two species. As the former is a non-pathogen and *N. meningitidis* represents a pathogen of considerable importance in human health, exchanges of genetic material between them might have consequences for our understanding of their evolution. Moreover, the diversity and the extent of recombination indicated among the *N. meningitidis* populations highlight that it is necessary to consider these pathogens as a heterogeneous population, and that multiple pathways of evolution may arise among them as a response to treatment strategies, including antibiotics and vaccines, as also recently discussed in [31]. For details concerning the currently available Meningococcal vaccines, see, e.g. [32] and [33]. Contrary to some of the previous studies of recombination and population structure in Meningococci, e.g. [34]–[36]), where only very limited sample sizes were considered, we have here focused on the detailed exploration of a more extensive database using multiple model-based statistical tools. In summary, our combined results illustrate crisply the possibility of using large-scale MLST sequence data to draw attention to currents in the gene pool, i.e. specific populations that seem more likely to undergo recombination, including recombination with different species. More detailed exploration of such groups of strains could then shed new light on the mechanisms that shape the joint evolution of pathogens and non-pathogens sharing ecological niches.
